# Adamantinoma Metastasis to the Pelvis and Ovaries: A Case Report and Literature Review

**DOI:** 10.7759/cureus.50087

**Published:** 2023-12-06

**Authors:** Tarek Amin, Ayman Z Azzam, Saud K AlBatati, Abdullah M AlHossan

**Affiliations:** 1 Surgical Oncology Department, Oncology Center, King Faisal Specialist Hospital and Research Centre, Riyadh, SAU; 2 General Surgery Department, Faculty of Medicine, Alexandria University, Alexandria, EGY; 3 Orthopedic Surgery, King Fahad Military Medical Complex, Dhahran, SAU; 4 College of Medicine, Alfaisal University, Riyadh, SAU

**Keywords:** pelvis, lung metastasis, ovarian metastasis, metastatic adamantinoma, adamantinoma

## Abstract

Adamantinoma, constituting a minute fraction of primary bone tumors, poses a diagnostic challenge due to its ambiguous histogenesis. This report outlines a distinctive case involving a 27-year-old female with a history of right tibial adamantinoma, presenting with bilateral pulmonary emboli and metastasis to the ovaries and pelvic lymph nodes. Following en bloc resection five years earlier, the patient underwent debulking surgery with hyperthermic intraperitoneal chemotherapy (HIPIC) and intraoperative radiotherapy (IORT) as a palliative measure. The procedure achieved substantial pelvic tumor reduction, and subsequent follow-ups indicated a favorable postoperative trajectory. This case underscores the rarity of adamantinoma metastasis to the ovaries and pelvis, being the first reported instance, shedding light on the challenges and potential benefits of a multimodal palliative approach. Further research is warranted to refine treatment strategies for metastatic adamantinoma and enhance patient outcomes.

## Introduction

Adamantinoma, which constitutes only 0.1-0.5% of all primary bone tumours, is a rare low-grade malignant bone tumour of uncertain histogenesis [1]. The first reported example is attributed to Maier in 1900 [2]. Because of the lesion's uncanny histologic similarity to the jaw "adamantinoma" (ameloblastoma), Fischer, in 1913, named it "primary adamantinoma of the tibia"[3]. A unified histogenetic theory for the adamantinomas of the appendicular skeleton was put forth by Schulenberg in 1951 [4]. It mostly affects young individuals between the ages of 10 and 30.

The classical form and the differentiated type, which mimics osteofibrous dysplasia, are the two different types of adamantinoma [5]. Patients usually present with a firm, slowly enlarging mass that produces minimal disability. However, it can present rarely as a painful swelling or as a pathological fracture [6]. It may appear as a central or eccentric, multilocular, expansile, sharply or poorly delineated osteolytic lesion. The classical radiological finding is an area of lucency surrounded by sclerosis, usually involving the cortex and rarely the medullary cavity [7]. Osteofibrosis dysplasia (OFD) and fibrous dysplasia are included in the differential diagnosis of adamantinoma [7]. OFD occurs in the cortex without involving the medullary canal, whereas adamantinoma typically affects the anterior cortical bone and extends into the bone marrow [7].

Adamantinoma is best treated via broad excision with clear margins [8]. Curettage is not recommended due to the high rate of recurrence [9]. Chemotherapy and radiotherapy are not effective in the treatment of adamantinoma [9]. Adamantinoma has the potential to metastasize and is locally aggressive in nature [10]. In this case report, we present a rare case of adamantinoma that presented with bilateral pulmonary embolism and metastasis to the ovaries and pelvis.

## Case presentation

**Figure 1:**
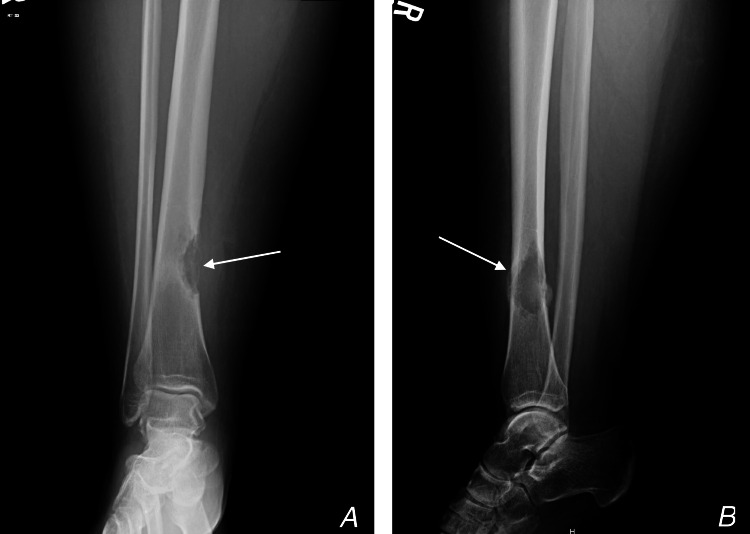
X-ray images of the patient A: Shows an anterior-posterior view of a right distal tibia, B: Shows a lateral view of the distal tibia. Arrows showing a well-circumscribed, cortical tumor with a multilobulated, osteolytic pattern.

A 27-year-old female who underwent en bloc resection for an adamantinoma in her right tibia five years prior was referred to our surgical oncology department at King Faisal Specialist Hospital & Research Center (KFSH&RC), This was a case of metastatic adamantinoma to the pelvis after the initial tumor resection, which first presented as a right distal tibial lesion with no evidence of metastasis as seen in Figure 1.

**Figure 2:**
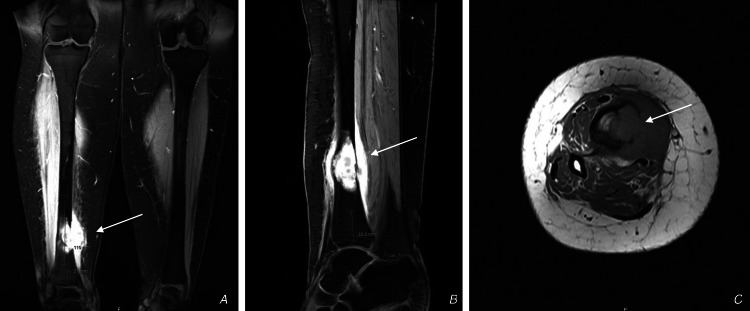
MRI images at the time of admission A: Shows a coronal cut of both tibias, B: Shows sagittal cut of the right distal tibia, C: Shows an axial cut of the right distal tibia. Arrows show the tumor invading the anterior and posterior deep muscle compartments of the right leg.

The patient underwent Magnetic Resonance Imaging (MRI) at the time of admission (Figure 2).

**Figure 3:**
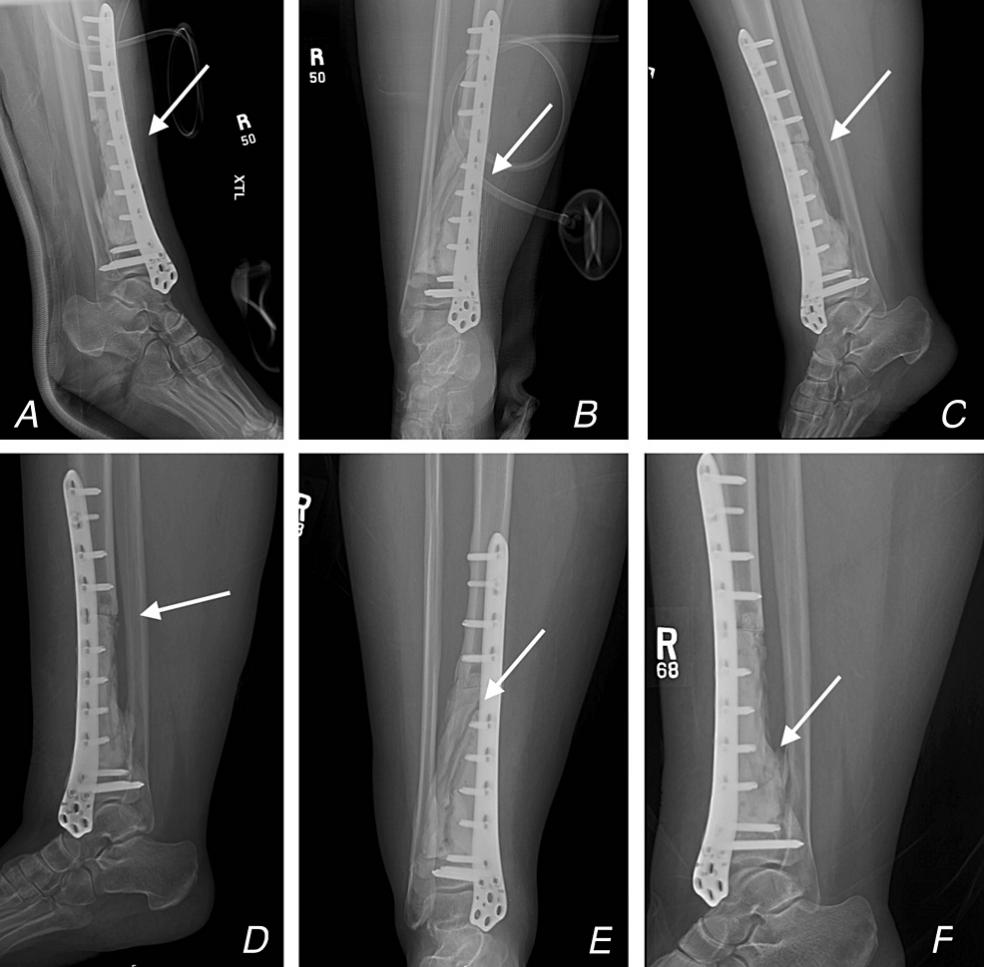
Post-operative X-ray images A: A lateral view of the right distal tibia and ankle X-ray on post-operation Day 1,  B: An antero-posterior (AP) view of the right distal tibia and ankle X-ray on post-operation Day 1,  C: Lateral view four months post-operation, D: Lateral view 10 months post-operation, E: AP view 14 months post-operation, F: Lateral view 14 months post-operation. Arrows showing fixation and the progressive bone cement integraion.

The MRI result suggested a high probability of an adamantinoma, in which an open incisional biopsy was obtained, which confirmed the diagnosis of adamantinoma. The patient underwent en-bloc resection with a clear surgical margin and reconstruction of bone loss with cement, a locked plate, and screws in 2016. The patient followed up with our orthopaedics clinic every three to six months with no complaints and serial chest and right tibia X-rays, as shown in Figure 3.

However, the patient missed three appointments and was lost to follow-up. The patient was referred back to our center from her local hospital in October 2021 following a CT scan of the chest, abdomen, and pelvis (CAP), which revealed a large pelvic mass requiring further investigations. Her initial presentation at the local hospital included symptoms of acute shortness of breath, abdominal pain, constipation, and a significant, unintentional weight loss of 35 kilograms over the preceding three months.

The patient was diagnosed with bilateral pulmonary emboli, which required admission to the intensive care unit for one week. After being treated with therapeutic anticoagulants and supportive measures, the patient underwent a CT scan and discovered the pelvic mass.

**Figure 4:**
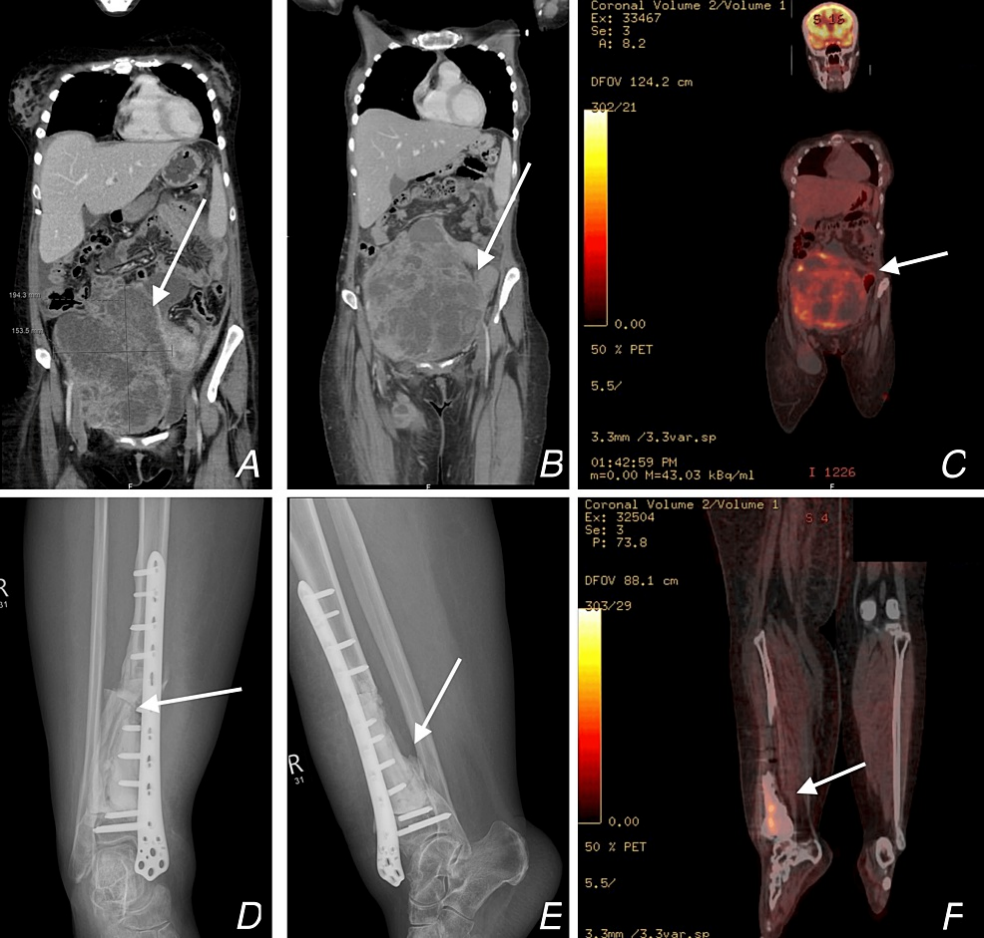
CT CAP images A: Coronal view of CT CAP measuring a pelvic mass of about 19.4 cm x 15.3 cm, pointed at by the arrow. B: Coronal view of CT CAP  showing the pelvic mass, pointed at by the arrow.  C: PET scan of the abdomen and pelvis, arrow showing the mass uptake of fluorodeoxyglucose. D: Antero-posterior (AP) view of the right distal tibia, the arrow of the distal tibial shaft displaced fracture of the cement. E: Lateral view of the right distal tibia, the arrow shows the distal tibial shaft displaced fracture of the cement. F: PET scan of the right tibia shows uptake of fluorodeoxyglucose. CT CAP: Computed tomography of chest, abdomen and pelvis, PET: Positron emission tomography.

Upon admission to our hospital under the internal medicine team. The patient's examination showed that her right lower limb had severe swelling, reaching up to the thigh. Abdominal examination revealed a massive mass that was palpable, hard, fixed, and non-tender. She underwent right distal tibia X-rays, CT CAP, and positron emission tomography (PET) scans (Figure 4). These scans revealed a large right adnexal complex mass lesion with mild free ascites and a partially thrombosed right superficial femoral vein, bilateral chronic pulmonary embolism, and an extraperitoneal large pelvic mass causing severe mass effects on the pelvic organs, corresponding most probably to right external iliac lymph node metastasis, also with other suspicious iliac lymph nodes.

The patient underwent a pelvic mass biopsy and nephrostomy tube insertion, and the biopsy revealed metastatic adamantinoma. The patient was referred to our surgical oncology department, where the decision was made that the patient would benefit mostly from a debulking surgery.

On March 27th, 2022, the patient underwent debulking surgery plus HIPIC (hyperthermic intraperitoneal chemotherapy) plus IORT (intraoperative radiotherapy). Intraoperatively a midline abdominal incision facilitated access to a highly vascular pelvic tumor, indicative of substantial venous return obstruction and resultant collateralization. Throughout the procedure, continuous hemostasis was maintained, A series of surgical interventions were performed: appendix resection, ovarian vessel ligation, and significant debulking, predominantly on the left side of the pelvis, enabling the successful excision of approximately 95% of the tumor. Intraoperative Radiation Therapy (IORT) was administered to the bladder tumor using a 6.5 cm applicator at a 30-degree angle, with a 0.5 cm bolus, followed by irradiation of the right pelvic sidewall, ensuring no overlap through demarcation with methylene blue, utilizing a 10 cm, 30-degree applicator. The skin and retractor were elevated to facilitate bidirectional Hyperthermic Intraperitoneal Chemotherapy (HIPEC) with ifosfamide, mesna, cisplatin, and doxorubicin. Subsequent to a 90-minute completion, additional oozing in the pelvic area was managed and controlled, with bilateral internal iliac ligation performed to address further oozing, noting the non-comparative positioning of the right and left internal iliac. Hemostatic material and tissue glue were applied, the omentum was mobilized to shield the pelvis and prevent bowel interaction with the tumor area, and two JP drains were inserted into the abdomen for pelvic drainage before concluding with standard mass closure of the wound.

**Figure 5:**
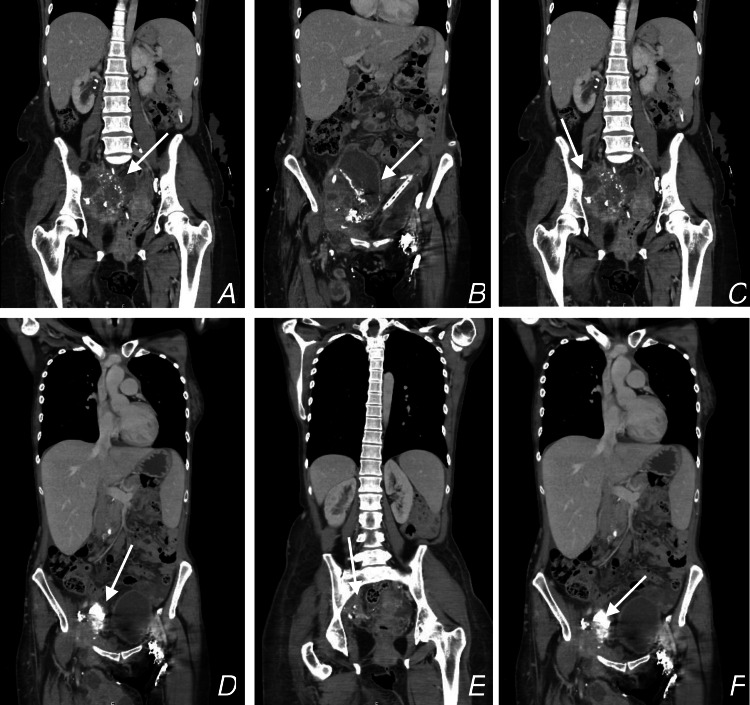
CT CAP images after debulking surgery A-E: Multiple coronal cuts of the CT CAP show the difference in tumor size before and after the debulking surgery. Arrows show an interval decrease in the size of pelvic sidewall tissue mass and an interval decrease in the size of right-inguinal metastatic lymph node with an increase in the size of areas of necrosis.

The patient was subsequently discharged 10 days post-operation in a stable condition without complications related to the surgery. The patient has since been under the follow-up care of our team, the palliative team, and the urology and oncology teams as an outpatient. The patient's CT scan, conducted four months postoperatively, is illustrated in Figure 5. The patient continues to be followed up in palliative care till 5 November 2023.

## Discussion

The origin of adamantinoma, a tumor with a controversial genesis, is characterized by its infrequent metastasis, low-grade nature, and local aggressiveness, as indicated by previous studies [1]. The long bones, particularly the tibia (80-85% of cases), are the tumor's primary site [3]. Other bones that are occasionally affected, in decreasing order of frequency, include the humerus, ulna, femur, fibula, radius, innominate bone, rib, spine, and rarely small bones of the hand and foot [11].

Adamantinoma has an enigmatic origin. A traditional hypothesis suggests the early migration of the skin's basal epithelial cells during embryonic development [12]. This hypothesis is supported by the observation that adamantinoma involvement is most common in the anterior tibia, where the enchondral-formed bone is closest to the skin's surface [6].

Two distinct histopathological types of adamantinoma exist. The first, known as classic adamantinoma, comprises both osteofibrous and epithelial components, typically affecting individuals over 20 years old and recognized for its low malignant potential [12]. The second type, differentiated adamantinoma, also called juvenile or osteofibrous dysplasia-like adamantinoma, predominantly affects patients under the age of 20. In this variant, histology reveals an osteofibrous dysplasia (OFD)-like pattern characterized by the absence of prominent nests and masses of epithelial cells, along with scarred cytokeratin positivity in the epithelial elements, making this type often benign [12].

On radiographs, adamantinoma often appears as a mono or multiloculated osteolytic lesion that is well-circumscribed with septa and a peripheral condensation [13]. The standard workup for adamantinomas involves a CT scan, which is useful for detecting pulmonary metastases [13]. MRI is essential for accurate locoregional staging, particularly for depicting distant cortical foci, soft tissue, and intramedullary extension, and is thus helpful for defining tumor-free margins [14,15]. The treatment of choice for adamantinoma is en bloc resection [8]. Marginal excision of the tumor can lead to a delayed local recurrence [8]. Following en bloc resection, distraction allografts, osteogenesis, non-vascularized autogenous bone grafts, vascularized autografts, and metallic segmental replacement can be performed for limb reconstruction [16].

Adamantinoma has unpredictable biological behavior. Even with a wide-margin excision, local recurrence and metastasis could occur [9]. According to a few studies in the literature, local recurrence occurs in 30-35% of patients, with a mortality rate of 6-18%, and lung metastases or lymph node involvement in 12-29% of cases [8,10]. Approximately 15-30% of adamantinoma cases result in lymphatic or hematogenous metastases to other parts of the body, most frequently the lungs or lymph nodes but less frequently the bones and abdominal viscera [16]. According to the Moon et al. report, 29 sites of clinical metastases were present at the time of death in 21 of the patients; 16 of these sites showed lung metastases, and five were found in lymph nodes [15]. Among 85 cases of adamantinoma in the study by Keeney et al., 31% of patients had local recurrence, 15% had lung metastases, and 7% had lymph node involvement [8].

Our patient presented with metastasis after four years of primary surgery. Metastasis to the ovaries is extremely rare. Hence, the presented case is unique in that the patient had extensive ovarian and pelvic lymph node metastasis. To the best of our knowledge, this is the first reported case of adamantinoma metastasis to the ovaries. A pelvic mass biopsy confirmed the diagnosis of metastatic adamantinoma.

The treatment of metastatic adamantinoma is challenging, and the prognosis is poor. It is estimated that patients with metastatic long-bone adamantinoma survive on average for 13 years [16]. In our case, the patient underwent debulking surgery with HIPIC (hyperthermic intraperitoneal chemotherapy) and IORT (intraoperative radiotherapy) as a palliative treatment aimed at reducing the tumor burden and improving symptoms [17]. HIPIC involves the delivery of heated chemotherapy directly into the peritoneal cavity during surgery to target any remaining cancer cells [18]. IORT, on the other hand, delivers a high dose of radiation directly to the tumor site during surgery to kill any remaining cancer cells [19]. In some studies, metastatectomy for pulmonary lesions has been described with good outcomes in both curative and palliative settings [20].

It’s worth noting that the effectiveness of debulking surgery, HIPIC, and IORT in treating adamantinoma metastasis is not well established, as there is limited research on the topic. Therefore, it’s important to weigh the potential benefits and risks of these treatments. We believe that this case study provides us with valuable insights into the topic and highlights the importance of regular follow-up visits for the early detection of metastatic adamantinoma.

## Conclusions

In conclusion, we report a rare case of adamantinoma metastasis to the pelvis and ovaries, which highlights the importance of considering metastatic disease in the differential diagnosis of pelvic mass in a young female with a history of adamantinoma. The case also emphasizes the role of debulking surgery as a form of palliative treatment in selected cases of metastatic adamantinoma and the value of prompt follow-up and monitoring for adamantinoma patients in order to identify and treat any complications as soon as they arise. Further studies are needed to establish the optimal treatment approach for metastatic adamantinoma and to improve the prognosis of this rare and aggressive tumor.
